# COVID-19 and renal infarct: To be or not to be on anticoagulation 

**DOI:** 10.5414/CNCS110602

**Published:** 2021-11-09

**Authors:** Chelsea Takamatsu, Paola Devis, Ramin Tolouian

**Affiliations:** 1 Department of Medicine, College of Medicine, University of Arizona,; 2 Department of Radiology, Southern Arizona VA Health Care System, and; 3 Renal Section, Southern Arizona VA Health Care System, College of Medicine, University of Arizona, Tucson, AZ, USA

**Keywords:** COVID-19, anticoagulation, renal infarction, spleen infarct, arterial thrombi, pulmonary emboli, Castleman disease

## Abstract

We present a unique case of a male veteran with a history of Castleman disease, presenting with multiple arterial and venous vascular thromboses in the setting of recent Coronavirus (COVID-19)-disease diagnosis. We explore this patient’s morbidity related to thrombotic complications of his COVID-19 diagnosis that were potentially avoidable with a comprehensive outpatient evaluation of his risk for thrombosis, as well as the initiation of anticoagulation and/or antiplatelet therapy given his high risk. Our case highlights the need for a standardized clinical workup of patients in the outpatient setting for risk assessment of vascular thrombosis associated with COVID-19 infection to direct medical management, in order to minimize adverse outcomes, complications requiring inpatient admission, and the need for additional yet limited medical resources and interventions. We propose a minimum of low-dose aspirin 81 mg daily as a reasonable approach for outpatient clinicians to consider, based on their best clinical judgement, when managing mild COVID-19, while other options, such as novel oral anticoagulants, are undergoing further investigation.

## Introduction 

Coronavirus disease 2019 (COVID-19) is characterized by a range of clinical presentations, mainly mild acute respiratory disease, which in 20% may lead to severe viral pneumonia with respiratory failure [1]. Soon after the start of the COVID-19 pandemic, it became very clear that SARS-CoV-2 was not only affecting the respiratory system, but could affect many other organs via the activation of numerous cytokine cascades [2]. One of the most dangerous and problematic issues was thrombosis formation in the vascular system. The incidence of thrombotic events in COVID-19 has been reported at 27% for venous vascular events and 3.7% for arterial vascular events [3]. Prophylactic anticoagulation of patients with COVID-19 in the hospital setting reduces the 30-day mortality by 27% [4]. 

## Case report 

A 71-year-old active male with emphysematous bronchitis requiring chronic home supplemental oxygen presented to the Emergency Department (ED) after a positive COVID-19 nasopharyngeal swab. He was hemodynamically stable without increased oxygen requirements from his baseline. He got discharged home with 3 days of dexamethasone 8 mg daily. He completed the course of steroids as prescribed. One week later, our patient returned to the ED after 3 hours of sharp, constant abdominal pain, most severe in the left upper quadrant, without any identifiable inciting event. He was tolerating oral intake that morning, had a normal bowel movement, and no changes in his urine output. He denied fevers, chills, skin rashes, worsening shortness of breath or cough, chest pain, urinary or GI symptoms. 

Past medical history was significant for unicentric Castleman disease of the right neck, which had undergone surgery many years prior and had since been inactive. The patient had also been diagnosed with a right lower extremity deep vein thrombosis (DVT) 5 months prior and took only 3 months of rivaroxaban 20 mg daily and was not on any prophylactic anticoagulation over the 2 months prior to this visit. He denied a personal history of atrial fibrillation. His home medications consisted of lamotrigine, citalopram, bupropion, fluticasone nasal spray, as-needed tylenol for pain, and a daily multivitamin. He also denied a sedentary lifestyle or recent travel. Social history was negative for active tobacco, alcohol, or recreational drug use. There was no family history of clotting disorders. 

On this second ED presentation, the patient was fully oriented and in no acute distress, without any skin rash or edema. Cardiovascular exam was benign. Mild diffuse coarse breath sounds bilaterally were appreciated on lung auscultation. Abdominal exam noted a soft, non-distended abdomen with left upper quadrant tenderness to palpation with peritoneal signs. No abdominal bruits were detected. 

**Figure 1:**
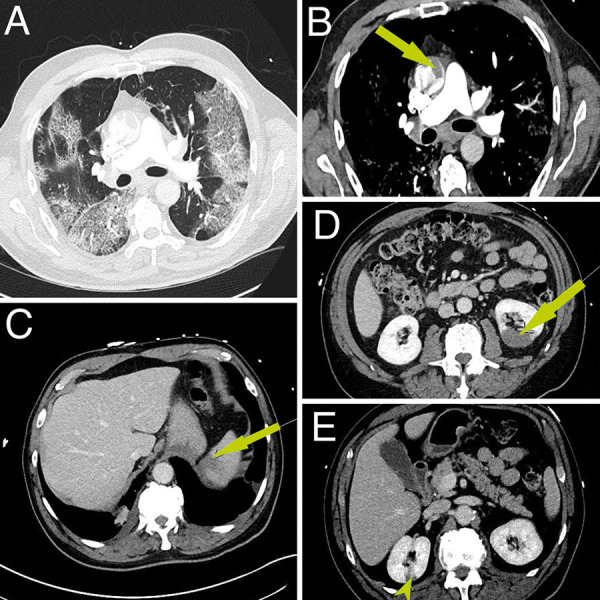
A: Bilateral, peripheral, ground-glass opacities diffusely involving the lungs, consistent with the diagnosis of moderate COVID-19 pneumonia. B: Arrow points to a large adherent thrombus on the anterior wall of the ascending thoracic aorta. C: Arrow points to a superior pole splenic infarction. D: Arrow points to a large infarct in the posterior cortex of the left kidney. E: Arrow points to a small infarct in the posterior cortex of the right kidney.

ED labs were significant for a white blood cell count of 9.9 × 10^3^/μL with neutrophilia to 8.3 × 10^3^/μL K and lymphopenia to 0.7 × 10^3^/μL on differential, new thrombocytosis to 510 × 10^3^/μL, elevated alanine amino transferase (ALT) of 76 U/L, and alkaline phosphatase to 146 U/L. Urinalysis was negative for hematuria. Pro-BNP was elevated to 1,010. Additional inpatient labs were notable for elevated serum C-reactive protein (CRP), IL-6 to 16.26 pg/mL, and reduced protein S activity (Table 1). EKG showed normal sinus rhythm without acute ischemic changes. Computed tomography angiography (CTA) chest and computed tomography (CT) abdomen/pelvis demonstrated bilateral peripheral ground-glass opacities with multifocal consolidations consistent with moderate COVID-19 pneumonia, an ascending aortic mural thrombus, subsegmental pulmonary emboli (PE), and infarcts in the spleen and bilateral kidneys (Figure 1). 

In the ED, our patient was started on a heparin drip and morphine for pain relief. He was admitted to the COVID-19 ICU for further management. A Doppler ultrasound of lower extremities was negative for DVT. A complete transthoracic echocardiogram (TTE) did not show any intracardiac thrombi in the left ventricle or valvular vegetations but identified a small patent foramen ovale (PFO) and noted mid-range ejection fraction of 45 – 50%. Interventional Cardiology deferred cardiac catheterization, due to the risk of distal embolization. Hematology recommended starting baby aspirin and transitioning from the heparin drip to apixaban 5 mg twice daily for 6 months. 

On day 10 of hospitalization, the hospital course was complicated by the development of a large right pneumothorax, requiring a chest tube. After a 19-day hospital admission, our patient was discharged to a skilled nursing facility in stable condition. 

## Discussion 

The leading cause of arterial emboli is atrial fibrillation, followed by aortic atherosclerotic plaque [5]. Our patient did not have any cardiac arrhythmia, and his small PFO was never problematic in his 7 decades of life before COVID-19 illness. This was a rare presentation of a patient with mild to moderate pulmonary symptoms of COVID-19, but emergent needs for hospital admission due to multi-system infarcts from systemic hypercoagulability, primarily triggered by SARS-CoV-2 virus. The pathogenesis of hypercoagulability in COVID-19 is complex. Numerous studies support increased venous and arterial thrombotic incidents due to clotting cascade derangements, widespread cytokine release, direct cytopathic effects on endothelial cell, complement activation, thrombin generation related to SARS-CoV-2 virus and prothrombotic antibodies, in the context of local immune response [6, 7, 8]. 

Our patient had a pre-existing coagulopathy with a recent history of DVT several months prior to his COVID diagnosis. He also had a history of unicentric Castleman disease of a lymph node (hyaline vascular histopathologic subtype) in the neck, which is a rare lymphoproliferative disorder that typically carries a good prognosis. Although it seems Castleman disease was inactive, it has been observed that IL-6 elevation (in our case) is associated with increased thrombosis risk, which carries a worse prognosis if arterial thrombosis occurs [9]. 

A literature review identified a total of 3 published cases of concomitant renal and splenic infarcts in COVID-19 patients like our case. The consensus was to consider DVT/PE or infarction of abdominal viscera when a patient presents with chest or abdominal pain several days after COVID-19 diagnosis [10]. 

To this date, there are no documented cases of COVID-19 patients presenting with multiple arterial thrombi to the extent of our patient, who not only had bilateral renal infarcts, but also a splenic infarct, pulmonary embolism, and aortic arch mural thrombus. Kundal et al. [11] described a young female with a descending aortic thrombus proximal to the diaphragmatic hiatus, smaller mural thrombi proximal to the bilateral kidneys, and unilateral embolic infarct of the right kidney. However, this patient had predisposing risk factors of a moderate-sized PFO, oral contraceptive use, and poorly controlled hypertension. Our patient had a small PFO noted on TTE, but never experienced arterial thrombotic events until his COVID-19 diagnosis. Our study suggests the need to maintain higher clinical suspicion for arterial and venous thrombosis in COVID-19 patients and to consider prophylactic management in these patients. 

Currently, there are numerous guidelines based on expert opinions comprising the American Society of Hematology, NIH, and CHEST, providing recommendations for standard prophylactic doses of anticoagulation in all hospitalized COVID-19 patients. In critically ill patients with COVID-19, escalated doses of thromboprophylaxis have been recommended. Although no significant differences in death and thrombotic events have recently been shown in patients with COVID-19 who were treated with standard- vs. intermediate-dose heparin-based thromboprophylaxis [12, 13]. 

Highlighted by these COVID patients who experienced minimal respiratory symptoms, questions arise as to whether these emergent macrovascular arterial thromboses were preventable with individualized outpatient anticoagulation and/or antiplatelet therapy. Nonetheless, there are currently no definitive, evidence-based outpatient anticoagulation recommendations for patients with active, but asymptomatic or mild COVID infection. Moreover, timing and intensity of anticoagulation for prophylactic measures, especially in non-hospitalized patients with mild COVID-19, remains undetermined. 

In clinical practice, multiple biomarkers to foresee the probability of thrombotic events have been used with varying predicting values. For example, it has been proposed that IL-6 levels > 32.1 pg/mL, CRP > 41.8 mg/L, and D-dimer > 1,000 ng/mL might be a good predictor of thrombosis risk to guide prophylactic anticoagulation in patients with mild respiratory symptoms of COVID-19 in the outpatient setting [14, 15]. 

More studies are needed to explore the utility of CRP, D-dimer, and IL-6, and to determine what degree of elevation predicts prothrombotic risk. This is an area of potential promise for COVID-19 management, based on reduced mortality with anti-IL-6 inhibitor therapy [16]. D-dimer is widely available and a highly sensitive test for venous thrombosis. D-dimer has a high negative predictive value with normal D-dimer levels ruling out thrombotic events [17]. 

Likewise, our case suggests the need for a broader outpatient workup and individualized risk stratification for thrombotic complications of COVID-19, considering medical history of hypercoagulability and abnormal prothrombotic lab values, such as IL-6 and CRP. 

In outpatient settings, we suggest starting anticoagulation in patients with mild to moderate COVID-19 disease for 2 weeks if there is a history of previous DVT/PE, PFO, current use of hormone contraceptives, active malignancy, hypercoagulable states like Castleman disease, BMI > 30, or elevated D-dimer levels > 1,000 ng/mL. Currently, there is no consensus regarding the choice of anticoagulation medications. There is an ongoing clinical trial (ClinicalTrials.gov Identifier: NCT04746339) to assess the efficacy of apixaban 2.5 mg twice daily for prophylaxis of thromboembolic events in COVID-19, but until conclusions are drawn, there should be a reasonable short-term option to avoid thrombotic complications. 

In all other patients without any hypercoagulable risk factors or increased risks of adverse bleeding such as thrombocytopenia, low-dose aspirin 81 mg daily might be a reasonable choice, especially as data has reported the efficacy of aspirin in decreasing ICU admissions and mortality in patients with COVID-19 [18, 19]. 

## Acknowledgment 

Emergency Department of Southern Arizona VA Health Care System in Tucson, AZ, USA. 

## Funding 

None. 

## Conflict of interest 

The authors declare no relevant financial interests or conflict of interest. 

**Table 1.** Laboratory parameters with trends by day since initial hospitalization.

Laboratory parameter	Reference range	Admission	Hospital day 4	Hospital day 6	Hospital day 11	Discharge – LOS 19
Leukocytes (× 10^3^)	4.5 – 11	9.9	13.8 H			
Hemoglobin (g/dL)	14 – 16	13.3 L	11.1 L			
MCV (fL)	80 – 99	73.3	73.6 L			
Thrombocytes (× 10^3^)	150 – 400	510 H	447 H			
Sodium (mEq/L)	135 – 145	133 L		133 L	132 L	138
Potassium (mEq/L)	3.5 – 5.5	3.9		5.8 H	4.2	4.5
Chloride (mmol/L)	98 – 107	104		96 L	99	99
Bicarbonate (mEq/L)	21 – 29	21		29	27	31 H
Blood urea nitrogen (mg/dL)	5 – 20	21 H		21 H	21 H	23 H
Serum creatinine (mg/dL)	0.7 – 1.4	0.7		0.7	0.6 L	0.8
Estimated glomerular filtration rate (mL/min/1.73m^2^)		111		111	133	111
Glucose (mg/dL)	60 – 105	106 H			134 H	81
Anion gap	3 – 14	8		8		8
Magnesium	1.5 – 2.6	2				
Lipase (U/L)	25 – 250	38				
Total bilirubin (mg/dL)	0.1 – 1.2	1.3 H				
Aspartate aminotransferase (U/L)	15 – 45	42				
Alanine aminotransferase (U/L)	5 – 40	76 H				
Alkaline phosphatase (U/L)	40 – 125	146 H				
Lactate dehydrogenase (U/L)					771 H	
CK (U/L)	25 – 175					
BNP (pg/mL)	0 – 125	1,010 H				
Serum osm (mOsm/kg)	284 – 306	280 L				
Coagulation	Reference range					
aPTT (s)	24.8 – 36.9	56.2 H				
PT (s)	12.1 – 14.2	15.2 H				
INR	1 – 1.1	1.2 H				
Immunologic analysis	Reference range					
Protein C activity (%)	70 – 180		132	123		
Protein S activity (%)	70 – 150		31 L	21 L		
Interleukin 6 (pg/mL)	0 – 5	16.26 H				
Factor V mutation	Wild type	Wild type		Wild type		
Factor II mutation	Wild type	Wild type		Wild type		

H = high; L = low.
